# Single-cell transcriptome-wide Mendelian randomization and colocalization analyses uncover cell-specific mechanisms in atherosclerotic cardiovascular disease

**DOI:** 10.1016/j.ajhg.2025.06.001

**Published:** 2025-06-23

**Authors:** Anushree Ray, Paulo Alabarse, Rainer Malik, Muralidharan Sargurupremraj, Jürgen Bernhagen, Martin Dichgans, Sebastian-Edgar Baumeister, Marios K. Georgakis

**Affiliations:** 1 Institute for Stroke and Dementia Research (ISD), Ludwig-Maximilians-University (LMU) Hospital, LMU Munich, 81377 Munich, Germany; 2 Glenn Biggs Institute for Alzheimer’s & Neurodegenerative Diseases, University of Texas Health Sciences Center, San Antonio, TX 78229, USA; 3 German Centre for Cardiovascular Research (DZHK), Partner Site Munich Heart Alliance, 80636 Munich, Germany; 4 Munich Cluster for Systems Neurology (SyNergy), 81377 Munich, Germany; 5 German Center for Neurodegenerative Diseases (DZNE) Munich, 81377 Munich, Germany; 6 Institute of Health Services Research in Dentistry, University of Muenster, 48149 Muenster, Germany; 7 Program in Medical and Population Genetics, Broad Institute of MIT and Harvard, Cambridge, MA 02142, USA

**Keywords:** atherosclerosis, cardiovascular disease, expression quantitative trait loci, RNA sequencing, Mendelian randomization, colocalization analysis, drug target, immune cells

## Abstract

Genome-wide association studies (GWASs) have identified numerous genetic loci influencing human disease risk; however, linking these to causal genes remains challenging, limiting opportunities for drug target discovery. Transcriptome-wide association studies (TWASs) address this by linking variants to gene expression but typically rely on bulk RNA sequencing, limiting cell-specific resolution. Here, we present a single-cell TWAS pipeline combining *cis*-Mendelian randomization (MR) with colocalization analyses at the single-cell level. As a case study, we examined how genetically proxied gene expression in immune cells influences atherosclerotic cardiovascular disease (ASCVD) risk. We integrated single-cell expression quantitative trait loci (sc-eQTLs) for 14 immune cell types with GWASs for coronary artery disease, large artery atherosclerotic stroke, and peripheral artery disease. sc-*cis*-MR revealed 440 gene-outcome associations across cell types, 88% of which were missed by bulk TWASs, despite the considerably smaller sample size of the sc-eQTL dataset. Of these associations, 21 were replicated with external *cis*-eQTLs and colocalized with ASCVD GWAS signals. Expanding on previous evidence linking genetically proxied *LIPA* expression in whole blood to coronary artery disease, we found genetic variants influencing *LIPA* expression, particularly in monocytes, to drive associations with coronary artery disease, large artery atherosclerotic stroke, and subclinical atherosclerosis traits. A phenome-wide association study confirmed these findings without evidence of associations with unexpected clinical outcomes. scRNA sequencing and immunohistochemistry of human carotid plaques revealed high *LIPA* expression in plaque macrophages. Our pipeline enables the discovery of cell-specific expression patterns that drive genetic predisposition to human disease, potentially impacting target selection for cell-tailored therapeutics.

## Teaser

We present a stringent single-cell analytical framework using transcriptome-wide Mendelian randomization and colocalization. By resolving cell-type-specific expression effects missed by bulk methods, this approach improves causal gene prioritization in atherosclerotic cardiovascular disease and offers a scalable strategy for uncovering disease mechanisms and therapeutic targets across diverse traits.

## Introduction

Analyses of human genetic data can provide invaluable insights into causal disease mechanisms and inform the development of new drugs.^1^ Indeed, drug targets with genetic support are more than twice as likely to deliver drugs that will be approved.^2^^,^^3^ In the field of atherosclerotic cardiovascular disease (ASCVD), signals from genetic studies have informed or contributed to the emergence of several drug development programs, including PCSK9 inhibitors,^4^ Lp(a)-lowering molecules,^5^ ApoC3- and ANGPTL3-targeting agents,^6^ factor XI inhibitors,^7^ and interleukin (IL)-6 signaling inhibitors.^8^ Genome-wide association studies (GWASs) have identified thousands of genomic loci associated with human disease.^9^ However, the translation of GWAS findings into actionable drug targets requires the determination of both causal genes regulated by the disease-associated variants and the specific cell types in which these causal genes exhibit their function.

Integrating GWAS data for clinical endpoints with data from other omics layers can provide valuable insights into causal genes for human disease at scale. For example, transcriptome-wide association studies (TWASs) use gene expression levels instrumented by *cis*-expression quantitative trait loci (*cis*-eQTLs) to identify tissue-specific and functionally relevant genes associated with disease outcomes from GWAS loci.^10^ However, gene expression is regulated at the cellular level and not the tissue level. As such, eQTLs could be specific to distinct cell types that are relatively rare in a given tissue and obscured in bulk analyses that average gene expression from diverse cell types. A higher-resolution characterization of the biological complexity and cellular heterogeneity of ASCVD could be obtained from single-cell omics technologies. Integration of single-cell transcriptome profiles from single-cell RNA sequencing (scRNA-seq) and GWAS data could enhance our current understanding of disease mechanisms, aid the identification of cell-specific druggable targets, and facilitate the development of tailored interventions, such as cell-targeted RNA therapeutics.

Here, we present a single-cell TWAS pipeline that combines *cis*-Mendelian randomization (MR) using cell-specific *cis*-eQTL variants alongside colocalization analyses to identify potential causal cell-specific expression changes underlying GWAS signals. As a case study, we integrated immune cell-specific eQTL data with GWAS summary statistics for the most common manifestations of ASCVD—coronary artery disease (CAD), large artery atherosclerotic stroke (LAS), and peripheral artery disease (PAD)—to explore potential cell-specific immune mechanisms involved in atherosclerosis. Our analysis revealed cell specificity for established causal genes as well as previously underrecognized causal signals that could not be captured using bulk TWAS analyses, uncovering both the specific cell types in which these genes might exhibit their effects, as well as the direction of their effects on ASCVD outcomes. Using cell-specific *cis*-eQTL data from external cohorts, we replicated significant findings. We further performed downstream experimental and computational analyses to investigate an association between higher genetically proxied *LIPA* expression in monocytes and atherosclerosis.

## Methods

### Study design

**Figure 1:**
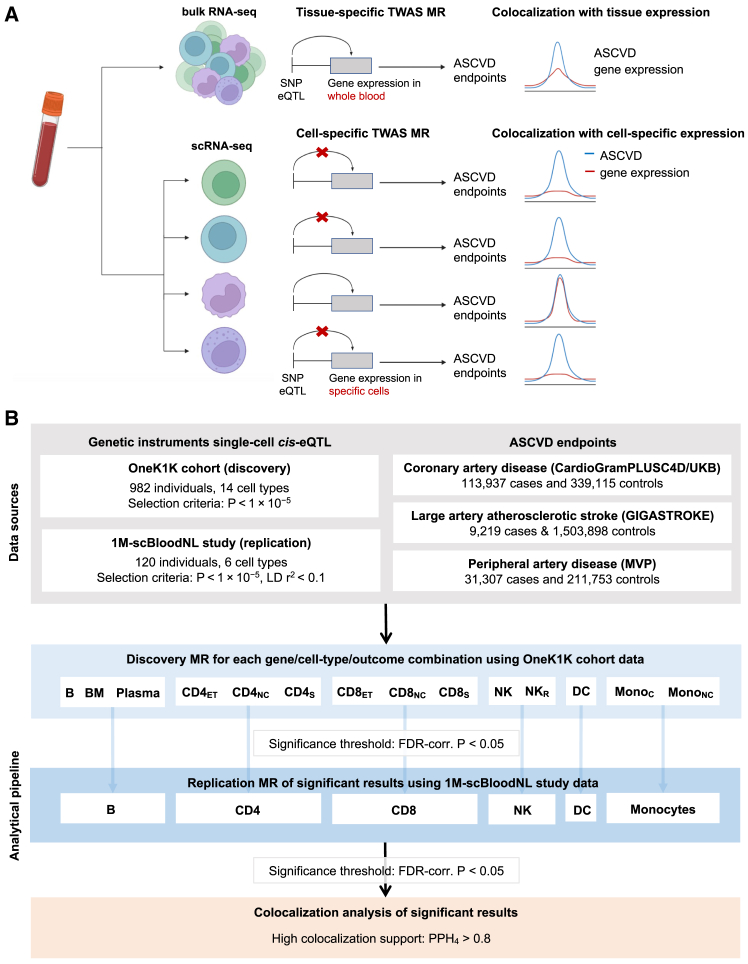
Study design (A) Schematic of our proposed approach for transcriptome-wide association studies using single-cell RNA sequencing (RNA-seq) vs. bulk RNA-seq data. Our approach includes Mendelian randomization analyses followed by colocalization. (B) Overview of the integrative genomic analysis pipeline and data sources used for this study. eQTL, expression quantitative trait loci; GWAS, genome-wide association study; CAD, coronary artery disease; LAS, large artery stroke; PAD, peripheral artery disease; MR, Mendelian randomization; B, immature and naive B cell; BM, memory B cell; CD4_NC_, CD4^+^ naive and central memory T cell; CD4_ET_, CD4^+^ effector memory and central memory T cell; CD4_S_, CD4^+^ SOX4 T cell; CD8_NC_, CD8^+^ naive and central memory T cell; CD8_ET_, CD8^+^ effector memory T cell; CD8_S_, CD8^+^ S100B T cell; NK, natural killer cell; NK_R_, natural killer cell recruiting; Mono_C_, classical monocyte; Mono_NC_, non-classical monocyte; DC, dendritic cell; FDR, false discovery rate; PPH, posterior probability hypothesis; TWAS, transcriptome-wide association study.

Our proposed pipeline for a single-cell TWAS is summarized in Figure 1. Briefly, similar to previous TWAS approaches at the bulk level,^11^^,^^12^^,^^13^ we used single-cell *cis*-eQTLs (sc-*cis*-eQTLs) derived from scRNA-seq studies as instruments for downstream MR analyses in GWAS summary data for outcomes of interest (discovery MR). As opposed to bulk RNA-seq, scRNA-seq datasets are usually smaller in scale. Therefore, to minimize false positive rates, we use an external dataset for the selection of sc-*cis*-eQTLs for the replication of the MR results (replication MR). Finally, to assess whether gene expression and cardiovascular outcomes shared the same causal variant rather than independent causal variants in high linkage disequilibrium (LD), we use colocalization analyses.

In our case study, we focused on exploring immune cell-specific mechanisms driving the risk of ASCVD, given the accumulating interest in the role of immune mechanisms in atherosclerosis.^14^ We started by leveraging cell-specific *cis*-eQTL data from peripheral blood mononuclear cells (PBMCs) in the OneK1K cohort (*N* = 982). In MR analyses, we then explored the effects of genetically proxied immune cell-specific gene expression on CAD (113,937 affected individuals and 339,115 control subjects), LAS (9,219 affected individuals and 1,503,898 control subjects), and PAD (31,307 affected individuals and 211,753 control subjects) using the largest available publicly available GWAS summary datasets. We replicated significant findings in sc-*cis*-eQTL data from the 1M-scBloodNL study (*N* = 120) and then applied colocalization analyses between *cis*-eQTL data from the OneK1K study and GWAS statistics for ASCVD phenotypes.

### Selection of genetic instruments

We obtained sc-eQTL mapping data, which integrate genotyping and scRNA-seq data, from the OneK1K cohort for the discovery analyses and from the 1M-scBloodNL for the replication analyses.^15^^,^^16^

The OneK1K cohort generated data from 1,267,758 PBMCs from 982 healthy individuals of Northern European ancestry.^15^ Based on transcriptional profile, 14 cell types were defined: B cell lineage was classified as plasma cells, immature and naive B cells, or memory B cells. CD4^+^ T cells were classified as naive and central memory T (CD4_NC_), effector memory and central memory T (CD4_ET_), and SOX4-expressing T (CD4_SOX4_) cells. Similarly, CD8^+^ T cells were classified as CD8_NC_, CD8_ET_, and CD8_SOX4_ cells. Innate immune lymphocytes were distinguished into natural killer (NK) and NK-recruiting cells, classical (Mono_C_) and non-classical (Mono_NC_) monocytes, and dendritic cells (DCs). For each gene/cell-type combination, *cis*-eQTLs were identified within a 1,000-kb region of either end of the gene. Summary statistics were available for all five SNPs for each gene/cell-type combination. For each gene/cell-type combination, we only used eQTLs reaching a *p* value threshold of <1 × 10^−5^, corresponding to a false discovery rate (FDR)-corrected *p* < 0.05 in a previous TWAS analysis.^10^

The 1M-scBloodNL study generated data from 928,275 PBMCs from 120 individuals from the Northern Netherlands population cohort Lifelines. sc-eQTL mapping data were available for six cell types based on marker gene expression: B cells, CD4^+^ T cells, CD8^+^ T cells, monocytes, NK cells, and DCs. For each gene/cell-type combination, summary statistics for associations of all SNPs within a 100-kb distance from the gene midpoint encoding the respective transcript were available for our analyses. We selected *cis*-eQTLs on the basis of an association at *p* < 1 × 10^−5^ and clumped them for LD using the clump_data function at a threshold of r^2^ < 0.1. As an alternative replication dataset, in sensitivity analyses, we leveraged summary statistics of bulk *cis*-eQTLs from fluorescence-activated cell-sorted (FACS) immune cells from 106 leukapheresis samples from Schmiedel et al.^17^ We used *cis*-eQTLs within a 1,000-kb region of each gene for six cell types—naive B cells, naive CD4^+^ T cells, naive CD8^+^ T cells, CD14^+^ CD16^–^ Mono_C_, CD14^–^ CD16^+^ Mono_NC_, and CD56^–^ CD16^+^ NK cells—based on associations at *p* < 1 × 10^−5^ and clumped at r^2^ < 0.1.

To compare our approach to a conventional TWAS, bulk eQTL summary statistics from 31,684 whole-blood samples of mostly European ancestry were obtained from the eQTLGen Consortium.^18^ We selected as genetic instruments *cis*-eQTLs within a 100-kb distance from the gene midpoint encoding the respective transcript that was associated with the levels of the respective transcript at *p* < 1 × 10^−5^. Thereafter, we clumped the genetic variants for LD at a threshold of r^2^ < 0.1.

### Clinical endpoints and intermediate phenotypes

We obtained trans-ancestry and European GWAS summary statistics for ischemic stroke and its subtypes from the GIGASTROKE GWAS meta-analysis of 86,668 affected individuals and 1,503,898 control subjects (67% European, East Asian, African, Hispanic, and South Asian ancestries).^19^ Of the individuals affected by ischemic stroke, 9,219 were subclassified as LAS. Summary statistics for CAD were obtained from a GWAS meta-analysis of 113,937 affected individuals and 339,115 control subjects of mostly (>95%) European ancestry conducted by Nelson et al.^20^ Summary statistics for PAD were obtained from a GWAS conducted in the Million Veteran Program (31,307 affected individuals and 211,753 control subjects) of European, African, and Hispanic ancestries (dbGAP under accession code dbGAP: phs001672.v2.p1).^21^ For follow-up analyses, we also used data for atherosclerosis endophenotypes—carotid plaque and coronary calcification. We obtained summary statistics from GWAS meta-analyses of cohorts of the CHARGE Consortium, including 48,434 individuals of European ancestry for carotid plaque (21,540 affected individuals and 26,894 control subjects) and 35,776 individuals of primarily (75%) European ancestry for coronary artery calcification score.^22^^,^^23^ GWAS meta-analysis summary statistics for CVD risk factors, including smoking status (1,232,091 European participants from GWAS and Sequencing Consortium of Alcohol and Nicotine Use), low- (LDL-C) and high- (HDL-C) density lipoprotein cholesterol (1,654,960 participants with 80% European, East Asian, African, Hispanic, and South Asian ancestries from Global Lipids Genetics Consortium), systolic (SBP) and diastolic (DBP) blood pressure (1,028,980 European participants), body mass index (BMI) (457,756 European participants from UK Biobank [UKB]), waist-hip ratio (WHR) (458,349 European participants from UKB), and glycated hemoglobin (HbA1c) levels (437,749 European participants from UKB) were also obtained.^24^^,^^25^^,^^26^^,^^27^

### MR

We undertook a two-stage (discovery and replication) MR approach to systematically evaluate evidence for the putative causal effects of immune cell-specific gene expression on the six cardiovascular outcomes.^10^ The discovery MR analyses were conducted between *cis*-eQTLs in 14 immune cell types from the OneK1K cohort and CAD, LAS (trans-ancestry and European), and PAD using the TwoSampleMR R package (v.0.6.8).^28^ To ensure the identical orientation of effect alleles between the eQTLs and outcome associations, we harmonized the exposure and outcome datasets using the harmonise_data() function. Subsequently, if only a single eQTL was available for a gene, the Wald ratio estimate was obtained. If more than one SNP was available, the inverse-variance weighted (IVW) method was used to obtain an effect estimate. All *p* values were adjusted using the Benjamini-Hochberg method to control the FDR in multiple comparisons.^29^ For each outcome, the pairwise weighted Pearson correlation of the discovery MR analyses results between different cell types, as well as bulk-eQTL MR analyses results, were determined and visualized as a correlation matrix.

We brought forward significant target genes in the discovery MR analyses (FDR-corrected *p* < 0.05) to the replication MR analysis. We reclassified the 14 cell types from the OneK1K cohort to the less dimensional six cell types in the 1M-scBloodNL study—B cells, CD4^+^ T cells, CD8^+^ T cells, NK cells, monocytes, and DCs.

### Colocalization

For significant cell-type/gene/outcome MR associations (FDR-corrected *p* < 0.05) in the replication MR analyses, we additionally performed colocalization analysis to determine whether gene expression and cardiovascular outcomes shared the same causal variant rather than the variant being shared due to LD. Colocalization analysis provides the posterior probabilities (PPs) of five hypotheses: neither gene expression nor the outcome is associated with genetic variants in the region (H0), only gene expression is associated with a genetic variant in the region (H1), only the outcome is associated with a genetic variant in the region (H2), gene expression and outcome are both associated with the region but with different causal variants (H3), and gene expression and outcome are associated with the same causal variant (H4). If a single instrumental variable (IV) was used to perform MR, the coloc.abf function from the coloc R package (v.5.2.3) was used with default prior probabilities of p1 = p2 = 1 × 10^−4^ and p12 = 1 × 10^−5^. Significant colocalization was defined as PPH4 ≥ 0.8.^30^ If more than one IV was used to perform MR, the coloc.susie function was used to account for the potential of >1 shared causal variants, and the maximum PPH4 value across multiple credible sets was considered.^31^

### Phenome-wide association study

To test the association between genetically proxied *LIPA* expression in monocytes with the full range of clinical phenotypes and detect possible unexpected associations with unexplored phenotypes, we used DeepPheWAS and assigned 487,314 participants from the population-based UKB with standardized Phecodes representing disease entities.^32^ We used all ICD10 codes (main position, secondary position, and death records) from the UKB. We excluded Phecodes with <100 cases and Phecodes that are male or female specific, leading to a total of 1,312 phenotypes. Individuals were assigned a case status if >1 ICD10 code mapped to the respective Phecode. Individuals meeting the pre-specified exclusion criteria were removed from the analysis; otherwise, the individual was assigned a control status. We used logistic regression with age, sex, and 10 principal components as covariates to test variant carrier status (0/1) against the phenotype of interest. Wald ratio MR analyses were performed for genetically proxied monocyte *LIPA* expression (1 variant). Results reaching an FDR-corrected *p* < 0.05 were considered statistically significant.

### scRNA-seq analysis in human atherosclerotic plaques

To explore the expression of *LIPA* beyond whole blood in human atherosclerotic lesions, we downloaded individual-level scRNA-seq data from 15 carotid atherosclerotic plaques from Mocci et al. (Gene Expression Omnibus [GEO] accession number GEO: GSE260657).^33^ We analyzed the raw count matrices using the Seurat pipeline (v.5.1.0).^34^ In the initial preprocessing, we filtered out cells with fewer than 300 detected genes, those with total gene counts outside the range of 50,000 to 750,000, and cells with mitochondrial gene content exceeding 10% of total gene expression. Thereafter, we performed data normalization, variable feature identification, and scaling. To integrate data across samples, we selected common features and applied principal-component analysis (PCA) to each dataset. We combined datasets using integration anchors, followed by additional PCA and uniform manifold approximation and projection (UMAP) for dimensionality reduction and clustering. We annotated clusters by comparing cluster-specific marker genes with known cell-type markers and renamed cluster identities to accurately reflect cell types. A UMAP plot was generated to visualize the integrated data.

### Immunohistochemistry for LIPA in human atherosclerosis plaques

Carotid plaque samples were obtained from patients undergoing carotid endarterectomy at the Department of Vascular Surgery of the LMU University Hospital in Munich. The AtherOMICS Biobank has been approved by the ethics commission at LMU Munich (approval no. 22-0135), and the experiments were conducted according to the Declaration of Helsinki. Written informed consent was obtained from each individual. Following removal of the plaque, the carotid samples were fixed in 4% paraformaldehyde + 0.1 M phosphate buffered saline (pH 7.4) for 24 h, decalcified in EDTA (200 mM EDTA and 50 mM Trizma base [pH 8.0]), dehydrated, embedded in paraffin, and sectioned into 3.5-μm sections with a microtome. Plaque sections from three symptomatic individuals were used for staining. Slides were deparaffinized with Roti-Histol, then rehydrated progressively from 100% ethanol to distilled water, with a 5-min incubation in each step. Then, after antigen retrieval with trypsin for 10 min at 37°C, permeabilization in Tris-buffered saline (20 mM Trizma base and 200 mM sodium chloride [pH 7.6]) + 0.025% Triton X-100, pure cold methanol fixation for 10 min, and blocking with 2.5% normal horse serum for 20 min, the sections were incubated with primary antibodies against LIPA (1:50; PA5-97928, Thermo Scientific) and CD68 (1:100; 14-0681-82, Thermo Scientific) overnight at 4°C and fluorescent secondary antibodies (VectaFluor Duet Immunofluorescence Double Labeling Kit, DyLight 488 Anti-Rabbit, DyLight 594 Anti-Mouse, Vectorlabs) for 1 h at room temperature. Sections were mounted with DAPI (Abcam) mounting media with an antifade agent (Vectashield, Vectorlabs). Image acquisition was performed using a confocal microscope (LSM 980, Carl Zeiss), and images were recorded and processed with ZEN software (Carl Zeiss, v.3.3).

## Results

### Single-cell TWAS-MR uncovers cell-specific gene expression effects on ASCVD not captured by bulk TWAS-MR

Of the 6,468 genes analyzed in the OneK1K cohort, 5,162 (79.8%) had significant *cis*-eQTLs. The number of genes with significant *cis*-eQTLs varied widely across cell types, ranging from 4,411 for CD4_NC_ cells to 244 for plasma cells. For the majority of gene/cell-type combinations (81.7%), only a single *cis*-eQTL was retained as the instrument (Figure S1; Tables S1–S3). Between 7% and 61% of *cis*-eQTLs detected in individual cell types were not detected as *cis*-eQTL in bulk RNA-seq of whole blood in the much larger dataset of the eQTLGen consortium (*N* = 31,686 for bulk *cis*-eQTLs vs. *N* = 982 for sc-*cis*-eQTLs; Figures S2A and S2B).

**Figure 2:**
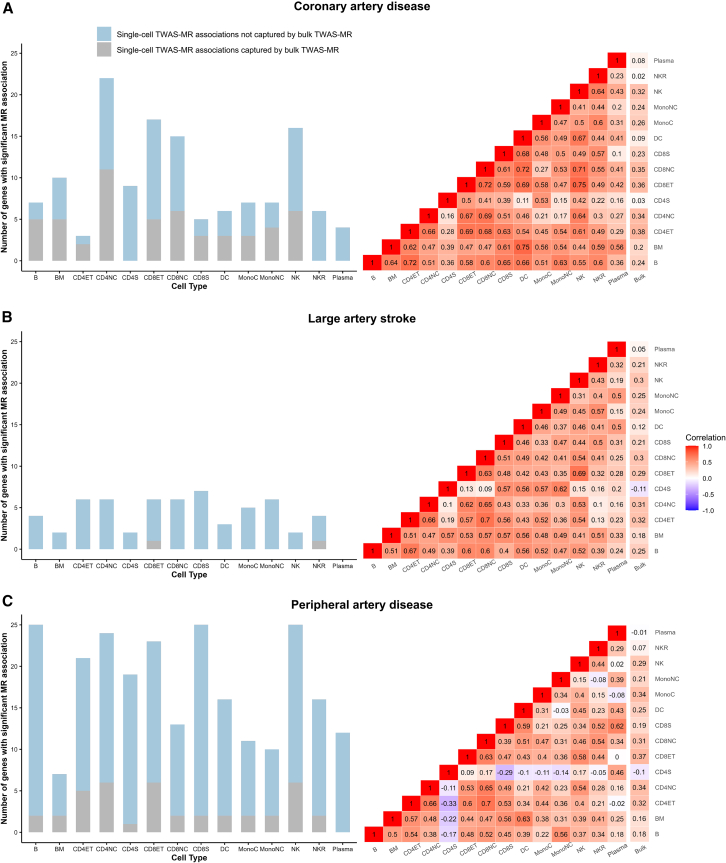
Comparison between results of single-cell and bulk Mendelian randomization analyses Stacked bar plot and correlation matrix of Mendelian randomization estimates for different immune cell types vs. whole blood for (A) coronary artery disease, (B) large artery atherosclerotic stroke, and (C) peripheral artery disease. B, immature and naive B cell; BM, memory B cell; CD4_NC_, CD4^+^ naive and central memory T cell; CD4_ET_, CD4^+^ effector memory and central memory T cell; CD4_S_, CD4^+^ SOX4 T cell; CD8_NC_, CD8^+^ naive and central memory T cell; CD8_ET_, CD8^+^ effector memory T cell; CD8_S_, CD8^+^ S100B T cell; NK, natural killer cell; NK_R_, natural killer cell recruiting; Mono_C_, classical monocyte; Mono_NC_, non-classical monocyte; DC, dendritic cell.

Detailed results of the discovery MR analysis (Wald ratio MR when the instrument consisted of a single *cis*-eQTL or IVW MR when the instrument consisted of >1 *cis*-eQTL) examining the relationship between genetically proxied cell-specific gene expression and ASCVD outcomes are shown in Tables S4–S6. Of 34,347 gene/cell-type/outcome combinations analyzed, 440 showed significant MR effect estimates (FDR-corrected *p* < 0.05), representing 318 unique gene-outcome pairs across different cell types. Notably, only 52 (16.4%; 32% for CAD, 3.9% for LAS, and 11.2% for PAD) of these significant associations were captured in MR analyses using bulk *cis*-eQTLs for whole blood, despite the much larger sample size used for *cis*-eQTL detection in bulk RNA-seq (*N* = 31,686 vs. *N* = 982; Figure 2). Furthermore, across all MR results, there were low to moderate correlations between bulk and single-cell effect estimates for all outcomes (median Pearson’s *r* for CAD: 0.49 [range: 0.02–0.75], for LAS: 0.43 [range: −0.11–0.7], and for PAD: 0.34 [range: 0.33–0.7]) (Figure 2). Collectively, these results indicate a significant gain in identified signals from the single-cell TWAS MR approach compared to bulk TWAS MR despite the considerably smaller sample sizes for sc-*cis*-eQTL discovery datasets.

### Replication of single-cell TWAS-MR and colocalization analyses

**Figure 3:**
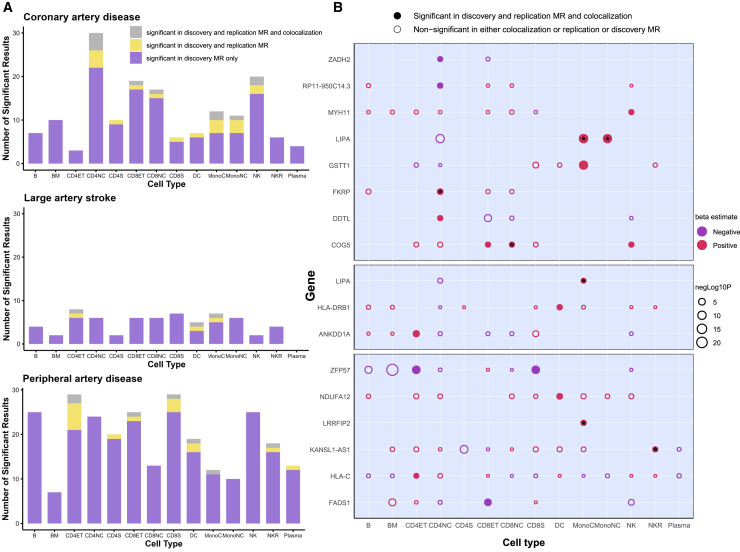
Associations between immune cell-type-specific gene expression and atherosclerotic cardiovascular disease outcomes (A) Stacked bar graphs of the number of genes whose cell-specific expressions were found to be significantly associated with coronary artery disease, large artery atherosclerotic stroke, and peripheral artery disease in each step of the statistical analysis. (B) Bubble heatmaps for cross-cell-type comparison of discovery Mendelian randomization (MR) estimates of cell-type/gene/outcome combinations, which had robust MR and colocalization evidence. Filled bubbles indicate associations that were significant in each of the three steps of the analysis pipeline. The color of the bubble corresponds to the beta coefficient of the association between the genetically predicted expression of genes (*y* axis) across different cell types (*x* axis) and the disease outcome. The size of each bubble corresponds to the negative logarithm of the discovery MR association false discovery rate-corrected *p* value. ^∗^ corresponds to cell-type/gene/outcome combinations that were also significant (FDR-corrected *p* < 0.05) in analyses using sorted cell-type-specific bulk eQTLs. B, immature and naive B cell; BM, memory B cell; CD4_NC_, CD4^+^ naive and central memory T cell; CD4_ET_, CD4^+^ effector memory and central memory T cell; CD4_S_, CD4^+^ SOX4 T cell; CD8_NC_, CD8^+^ naive and central memory T cell; CD8_ET_, CD8^+^ effector memory T cell; CD8_S_, CD8^+^ S100B T cell; NK, natural killer cell; NK_R_, natural killer cell recruiting; Mono_C_, classical monocyte; Mono_NC_, non-classical monocyte; DC, dendritic cell; ZADH2, zinc-binding alcohol dehydrogenase domain-containing protein 2; RP11-950C14.3, lncRNA, antisense to EIF2B2; MYH11, myosin heavy chain 11; LIPA, lipase A, lysosomal acid type; HLA, human leukocyte antigen; DDTL, D-dopachrome tautomerase like; COG5, component of oligomeric Golgi complex 5; NDUFA12, NADH:ubiquinone oxidoreductase subunit A12; LRRFIP2, leucine-rich repeat flightless-interacting protein 2; FADS1, fatty acid desaturase 1; LAS, large artery stroke; CAD, coronary artery disease; PAD, peripheral artery disease.

Of the 440 significant gene/cell-type/outcome combinations identified in the discovery MR, 38 achieved an FDR-corrected *p* < 0.05 in the replication MR analysis using genetic instruments from the 1M-scBloodNL study (Figure 3A; Tables S7 and S8). The two-stage MR analyses provided cell-specific causal effect estimates and directions of effect, which could help in further gene target prioritization and inform whether decreasing or increasing gene expression would be the desired effect in a translational context. Additionally, the stringency of colocalization analyses (PPH4 ≥ 0.8) led to enhanced prioritization of 21 gene/cell-type/outcome combinations—16 genes—with a high PP for a shared causal genetic variant (Figure 3A; Table S9). The effects of the 16 genes on the outcomes across different cell types are shown in Figure 3B. Sensitivity analyses using the analysis pipeline on GWASs for LAS in the European subpopulation prioritized the same gene/cell-type combinations as the trans-ancestry analysis (Table S10). Sensitivity analyses using bulk eQTL data of sorted cell types revealed that of 148 significant gene/cell-type/outcome combinations identified from discovery MR, 30 achieved an FDR-corrected *p* < 0.05 in the replication MR, with 19 also showing significant colocalization (Table S11). Of them, 7 signals overlapped with those significant in the sc-eQTL data analyses using the 1M-scBloodNL dataset (Figure 3B).

Although the genetically proxied expression of several genes, including *ZADH2*, *FKRP*, *COG5*, and *NDUFA12*, had directionally consistent effects, many genes had cell-specific effects. Specifically, higher *LIPA* expression in monocytes was associated with an increased risk of CAD and LAS, whereas higher *LIPA* expression in CD4_NC_ cells was associated with a lower risk of CAD and LAS. The results of MR analyses between the prioritized cell-type-specific gene expression and CVD risk factors are shown in Table S12. Notably, *LIPA* in Mono_C_ and Mono_NC_ cells was significantly associated with decreased LDL-C levels. Additionally, *LIPA* in Mono_NC_ cells was significantly associated with decreased HDL-C levels and increased SBP, DBP, and WHR.

### Monocyte-specific association between genetically proxied *LIPA* expression and atherosclerosis

**Figure 4:**
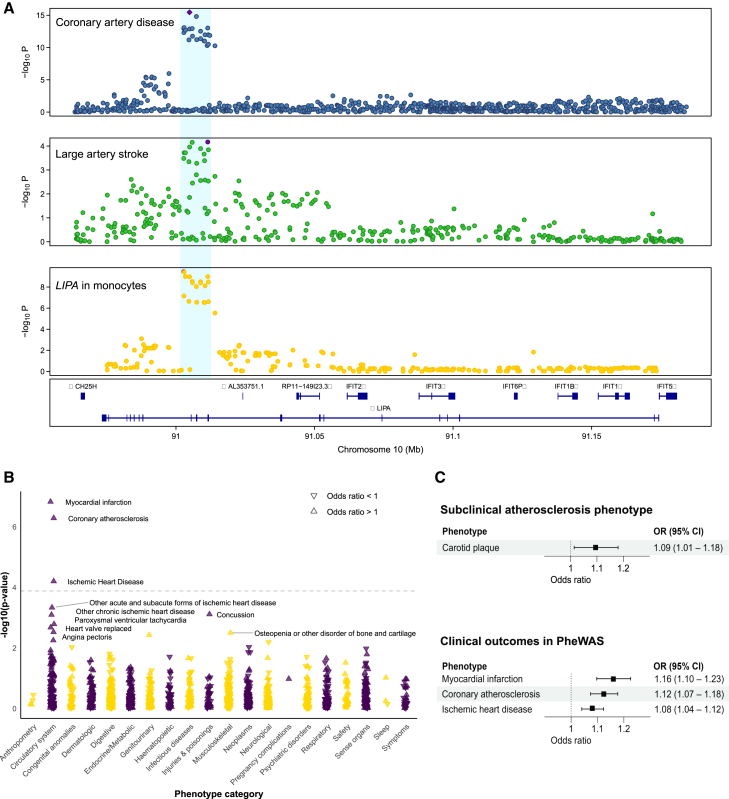
Association between genetically proxied monocyte expression of *LIPA* and atherosclerotic cardiovascular disease outcomes (A) LocusZoom plots illustrating evidence of genetic colocalization between *LIPA* expression in monocytes and coronary artery disease and large artery atherosclerotic stroke in the *LIPA* locus. (B) Forest plots of Mendelian randomization (MR) results for the effects of genetically proxied *LIPA* expression in monocytes and atherosclerotic cardiovascular outcomes. (C) Manhattan plot of an MR-phenome-wide association study for genetically proxied *LIPA* expression in monocytes. The dashed horizontal gray line represents a false discovery rate-corrected *p* value of 0.05.

While the association between *LIPA* eQTLs and CAD has been previously described in whole blood,^35^^,^^36^ our study adds evidence of colocalization between *LIPA* eQTLs and CAD and LAS GWAS association signals specifically in monocytes (Figure 4A). To further investigate this signal and explore associations with outcomes other than ASCVD, we performed a phenome-wide association study (PheWAS) analysis on 487,314 participants of the UKB. After correcting for multiple comparisons (FDR-corrected *p* < 0.05), the only phenotypes significantly associated with higher genetically proxied monocyte-specific *LIPA* expression in the PheWAS analyses were myocardial infarction, coronary atherosclerosis, and ischemic heart disease, thereby validating the relevance of *LIPA* in monocytes for ASCVD in an external dataset (Figure 4B; Table S13). There was no evidence of associations with other phenotypes in the opposite direction, supporting a favorable safety signal when genetically perturbing this drug target. Beyond clinical endpoints, we also found a significant association between genetically proxied monocyte *LIPA* expression and carotid plaque as captured by ultrasound, as well as myocardial infarction, ischemic heart disease, and coronary atherosclerosis (Figure 4C).

### mRNA expression and protein levels of *LIPA* in human atherosclerotic plaque macrophages

**Figure 5:**
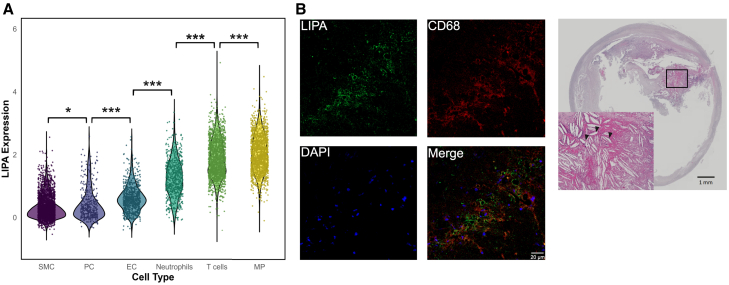
*LIPA* expression in human carotid atherosclerotic plaques (A) Violin plot of *LIPA* expression across different cell types in 15 human atherosclerotic plaque samples from Mocci et al.^33^*p* values of the Wilcoxon rank-sum test of expression between cell types are indicated as ^∗∗∗^*p* < 0.001, ^∗∗^*p* < 0.01, and ^∗^*p* < 0.05. (B) LIPA in macrophages (CD68) (scale bar, 100 μm) and cholesterol clefts (black arrowheads) (scale bar, 1 mm) in a human carotid plaque section from a symptomatic individual (*n* = 3). SMC, smooth muscle cells; PC, pericytes; EC, endothelial cells; MP, macrophages.

Given the evidence for an effect of monocyte-specific expression of *LIPA* on ASCVD, we, in a last step, examined whether *LIPA* is expressed in human atherosclerotic plaques and, more specifically, in plaque macrophages, which are primarily derived from circulating monocytes. Using published scRNA-seq data from 15 advanced human carotid artery plaques (Figure S3),^33^ we found *LIPA* to be expressed throughout all detected cell types, but its expression was highest in macrophages (Figure 5A). Accordingly, immunohistochemical staining of human carotid plaques from 3 individuals undergoing endarterectomy from the AtherOMICS cohort demonstrated LIPA in CD68-stained macrophages, along with abundant cholesterol clefts (Figures 5B and S4).

## Discussion

In the present study, we proposed and implemented an analytical pipeline for TWASs at the single-cell level. We demonstrated the applicability and potential of the approach by integrating sc-*cis*-eQTL data for 14 immune cell types in peripheral blood with GWAS data for three ASCVD outcomes—CAD, LAS, and PAD. Despite considerably smaller sample sizes of the sc-*cis*-eQTL discovery datasets, our single-cell MR analyses revealed significant information gains compared to bulk TWAS MR. Through our stringent screening criteria—including replication using an independent scRNA-seq dataset and genetic colocalization evidence—we identified 21 associations between cell-type-specific gene expression and ASCVD outcomes. Of these, the effects of *MYH11*, *LIPA*, and *FADS1* have been established previously in whole-tissue studies.^37^^,^^38^^,^^39^ Additionally, we identified previously underrecognized ASCVD risk associations of several genes, such as the CD4_NC_ cell-specific effect of the poorly characterized gene *DDTL*. While the expression of genes such as *COG5* and *ZFP57* was found to affect disease risk in multiple immune cell types, higher genetically proxied expression of *LIPA* was associated with a higher risk of two ASCVD outcomes—CAD and LAS—specifically in monocytes. Monocyte-specific *LIPA* also showed a strong association with lipid levels, blood pressure, and glycemic traits, suggesting broad metabolic regulatory roles. We validated the associations of monocytic expression of *LIPA* with atherosclerotic endophenotypes and clinical endpoints in PheWAS analyses in an external dataset, which also broadly supported a favorable safety profile with no significant signals for a higher risk of unexpected clinical outcomes. Finally, follow-up analyses of scRNA-seq data from human carotid plaques revealed high expression of LIPA in plaque macrophages, which was also confirmed at the protein level through immunohistochemistry.

Our approach enhances the conventional bulk TWAS-MR paradigm, enabling the detection of cell-specific expression patterns driving genetic predisposition to human disease. Since most genetic polymorphisms associated with human diseases are located in non-coding regions, it is believed that genetic variation influences predisposition to disease primarily by influencing gene expression patterns.^40^ In this context, TWAS approaches integrating GWAS findings with tissue-specific eQTL data have become crucial in post-GWAS explorations.^41^^,^^42^^,^^43^ As gene expression is regulated at the cellular level, using sc-eQTL data has significant benefits. Although scRNA-seq studies are still limited by sample size, we found that using cell-specific eQTL instruments enables the detection of signals that would not be detected with bulk eQTL instruments derived from much larger studies. This is reflected in the relatively weak correlation between cell-specific MR and bulk MR estimates for all coded genes across ASCVD outcomes. By integrating scRNA-seq eQTL data with GWAS risk loci using MR and colocalization analyses, we obtained evidence of potentially causal genes at risk loci for ASCVD and resolved specific cell types through which these genes exert their pathogenetic effects. For example, increased CD4_NC_ cell-specific *DDTL* expression was found to be associated with higher CAD risk, whereas increased Mono_C_-specific *LRRFIP2* expression was found to be associated with higher PAD risk. Our approach is generalizable to other outcomes, as well as single-cell and single-nuclei RNA-seq data from tissues beyond peripheral blood, as such data become increasingly available.

Higher genetically proxied monocyte expression of *LIPA* was associated with a higher risk of both CAD and LAS, highlighting its role in the development of atherosclerosis. Further, monocyte-specific *LIPA* expression was also significantly associated with LDL-C, HDL-C, SBP, DBP, and WHR, reinforcing its broader role in lipid metabolism and cardiometabolic risk. In a recent large genome-wide meta-analysis, the CAD and LAS lead variant at the LIPA locus, rs1412445, was associated with glycoprotein acetyl level—an established biomarker of chronic inflammation that has also been associated with the risk of ASCVD.^44^^,^^45^^,^^46^^,^^47^ Expanding multiomics integration to metabolomics could provide additional insights into how cell-specific gene expression contributes to ASCVD. The LIPA locus has been consistently identified as a risk locus for CAD in previous GWASs, with colocalization analyses suggesting *LIPA* as the causal gene at this locus.^48^^,^^49^ In multiple bulk eQTL datasets, the CAD lead variant at the LIPA locus, rs1412445, has been strongly associated with *LIPA* expression.^18^^,^^50^ CAD risk-enhancing alleles are also eQTLs for *LIPA* expression in whole blood.^51^ Our findings provide robust evidence that the effect of these genetic variants on *LIPA* expression in monocytes, rather than other PBMCs, drives the risk of CAD, LAS, and other atherosclerotic phenotypes. Consistently, rs1412445 and linked SNPs, rs1412444 and rs1320496, show strong enhancer activity in monocytes but not in CD4_NC_ cells, with PU.1 binding to the risk allele of rs1320496 enhancing *LIPA* expression specifically in monocytes, indicating differential SNP regulation across cell types.^38^ This aligns with a previous *in vitro* study in isolated human monocytes, which showed that risk-enhancing alleles increase not only *LIPA* expression but also the activity of the LIPA enzyme.^52^ In line with this, a study in *Ldlr* knockout mice has demonstrated that myeloid-specific *Lipa* overexpression leads to larger atherosclerotic lesions with higher macrophage content.^38^ These results suggest that the previously identified whole-blood association between *LIPA* expression and ASCVD could be primarily driven by monocyte-specific expression.

*LIPA* encodes lysosomal acid lipase, a key enzyme in lipid metabolism that hydrolyzes cholesteryl esters and triglycerides in lysosomes.^53^ Given that most atherosclerotic plaque macrophages originate from circulating monocytes, it is plausible that LIPA exerts its risk-enhancing effect by promoting excessive cholesterol crystal formation within these lesion macrophages. Supporting this hypothesis, our analysis revealed high LIPA mRNA and protein levels in macrophages within human atherosclerotic plaque samples with abundant cholesterol crystals. On the other hand, rare loss-of-function mutations leading to complete loss of LIPA activity and partial residual activity cause infant-onset Wolman disease and cholesteryl ester storage disease, respectively, with the latter also being associated with premature atherosclerosis, probably due to severe hyperlipidemia.^54^^,^^55^ This dual role—where reduced LIPA activity leads to hyperlipidemia-driven atherosclerosis and elevated LIPA expression may drive pro-inflammatory actions in macrophages—highlights the importance of LIPA homeostasis in preventing atherosclerosis. Reciprocally, the ability of our analysis pipeline to identify LIPA as an ASCVD risk driver, further supported by our PheWAS-MR and atherosclerotic plaque RNA-seq analysis, reinforces the robustness of our approach in detecting cell-specific genetic drivers of ASCVD risk.

The findings from our study could have implications for the development of RNA-based therapeutics, particularly those targeting gene expression in specific cell types. The identification of cell-specific gene expression patterns, such as the association of *LIPA* expression in monocytes and macrophages with ASCVD, underscores the potential for designing cell-tailored RNA therapies. RNA-based drugs are gaining pace in the cardiovascular field and becoming increasingly common, demonstrating the feasibility and effectiveness of these modalities. RNA-based drugs have been successfully introduced to clinical practice, such as inclisiran, a silencing RNA (siRNA) agent that targets the synthesis of PCSK9 to lower LDL-C,^56^ or are in advanced stages of clinical development, such as siRNA therapeutics against APOC3, ANGPTL3, Lp(a), and angiotensinogen.^57^^,^^58^^,^^59^^,^^60^ By focusing on modulating gene expression within specific cell types, it may be possible to mitigate disease risk while minimizing off-target effects. As RNA-based drugs continue to advance, our sc-TWAS MR pipeline provides a robust framework for *in silico* identification and validation of cell-specific targets, paving the way for more effective and safer therapies for complex diseases like ASCVD.

Our study has limitations. First, MR analysis at the single-cell level may miss risk genes with lower expression levels because the sparse expression in individual cells can limit statistical power, whereas bulk analysis averages gene expression across many cells, improving the detection of lowly expressed genes. Second, the relatively small sample size of scRNA-seq studies limited the number of genes tested and the number of eQTLs detected. The number of eQTLs obtained for each cell type from publicly available datasets varied according to the sample sizes for eQTL analyses of each cell type. Third, some eQTLs for very large genes in the 1M-scBloodNL study might not have been captured due to the *cis*-eQTL definition used of a 100-kb window centered around the gene midpoint. This might have led to the exclusion of eQTL-enriched promoter regions, thus explaining the low replication rate in this dataset. However, the median length of genes in the 1M-scBloodNL dataset is 27.4 kb, and over 80% of the genes are shorter than 100 kb. Considering promoter regions within 6 kb, a 100-kb window would capture the gene body and promoter region for most genes. Fourth, since most genes were associated with only one eQTL as a valid IV, sensitivity analyses to correct for horizontal pleiotropy could not be performed. The follow-up colocalization analyses provided evidence of a shared genetic basis between the exposure; however, they do not rule out the possibility that a single causal variant exerts pleiotropic effects on multiple neighboring genes.^61^ While alternative approaches, such as heterogeneity in dependent instruments (HEIDI), commonly used together with summary-data-based Mendelian randomization (SMR),^62^ could be applied instead of colocalization, we considered the more stringent nature of Bayesian colocalization to be better suited for our hypothesis-free transcriptome-wide pipeline.^63^ IVW was preferred over SMR, as it allows for the consideration of the effects of >1 variants, in cases where multiple independent variants influence cell-specific gene expression in a locus. Fifth, differences in scRNA-seq protocols could cause substantial variability in results. However, the consistency of the main findings across two independently collected scRNA-seq datasets raises confidence in their validity, although the finer resolution of cell types in the discovery dataset compared to the replication dataset may limit their direct comparability. Sixth, the GWAS and eQTL analyses in this study were primarily conducted on individuals of European ancestry, which may limit the generalizability of the findings to other ethnicities. Seventh, due to the lack of sc-eQTL data from human vasculature, we could not apply our transcriptome-wide approach to potentially more relevant tissues, where many of the ASCVD-associated variants might exert their effects.

In conclusion, we propose an integrative single-cell TWAS pipeline that could enhance our understanding of cell-specific gene expression patterns driving genetic predisposition to human disease. Using results from this approach as a foundation, we provided support for the key role of monocyte-specific *LIPA* in atherosclerosis with potential therapeutic relevance. Findings from single-cell TWASs could inform target selection for therapeutic modalities tailored to specific cell types, such as RNA therapeutics.

## Data and code availability

The published article includes all datasets generated during this study.

The complete single-cell transcriptome-wide Mendelian randomization and colocalization analysis pipeline is open source on GitHub (https://github.com/DeepVasc-Lab/3-step_sceQTLMR.git) under the Apache License 2.0.

## Acknowledgments

This work was funded by the 10.13039/501100001659German Research Foundation (DFG; Emmy Noether grant GZ: GE 3461/2-1, ID 512461526 to M.K.G. and Munich Cluster for Systems Neurology EXC 2145 SyNergy, ID 390857198 to M.K.G.), the Hertie Foundation (Hertie Network of Excellence in Clinical Neuroscience, ID P1230035 to M.K.G.), and the 10.13039/501100003390Fritz Thyssen Foundation (grant ref. 10.22.2.024MN to M.K.G.). J.B. acknowledges support from DFG grants SFB1123-A3 and Munich Cluster for Systems Neurology EXC 2145 SyNergy, ID 390857198.

## Author contributions

A.R. designed the study, performed the main analyses, and wrote the initial draft of the manuscript. P.A. contributed to immunohistochemistry. R.M. performed the PheWAS. M.K.G. designed and supervised the study and wrote the initial draft of the manuscript. All authors reviewed the manuscript and provided critical revisions.

## Declaration of interests

M.K.G. reports consulting fees from Tourmaline Bio, Inc., and serves on the editorial board of *Neurology*; both activities are unrelated to this work. J.B. is a co-inventor of patent applications covering anti-MIF strategies in inflammatory and cardiovascular diseases; this is unrelated to the current manuscript.

## Web resources

dbGAP, https://www.ncbi.nlm.nih.gov/gap/
GEO, https://www.ncbi.nlm.nih.gov/geo/

## Supplemental information

Data S1. Tables S1–S13

Document S2. Article plus supplemental information
