# Supplementary material for: Single-cell transcriptome-wide Mendelian randomization and colocalization analyses uncover cell-specific mechanisms in atherosclerotic cardiovascular disease
Source: Am J Hum Genet. 2025 Jun 23;112(7):1597–609. doi: 10.1016/j.ajhg.2025.06.001 (PMC12256824; doi:10.1016/j.ajhg.2025.06.001)

The American Journal of Human Genetics, Volume *112*

Supplemental information

Single-cell transcriptome-wide Mendelian randomization and colocalization analyses uncover cell-specific mechanisms in atherosclerotic cardiovascular disease

Anushree Ray, Paulo Alabarse, Rainer Malik, Muralidharan Sargurupremraj, Jürgen Bernhagen, Martin Dichgans, Sebastian-Edgar Baumeister, and Marios K. Georgakis

Supplemental Figures

Figure S1.

(a) Bar graph of the number of genes analyzed for each cell type for all outcomes in the discovery Mendelian randomization

(b) Stacked bar graph of the percentages of numbers of SNPs used in instrumental variables for discovery Mendelian randomization analysis of all gene/cell-type/outcome combinations

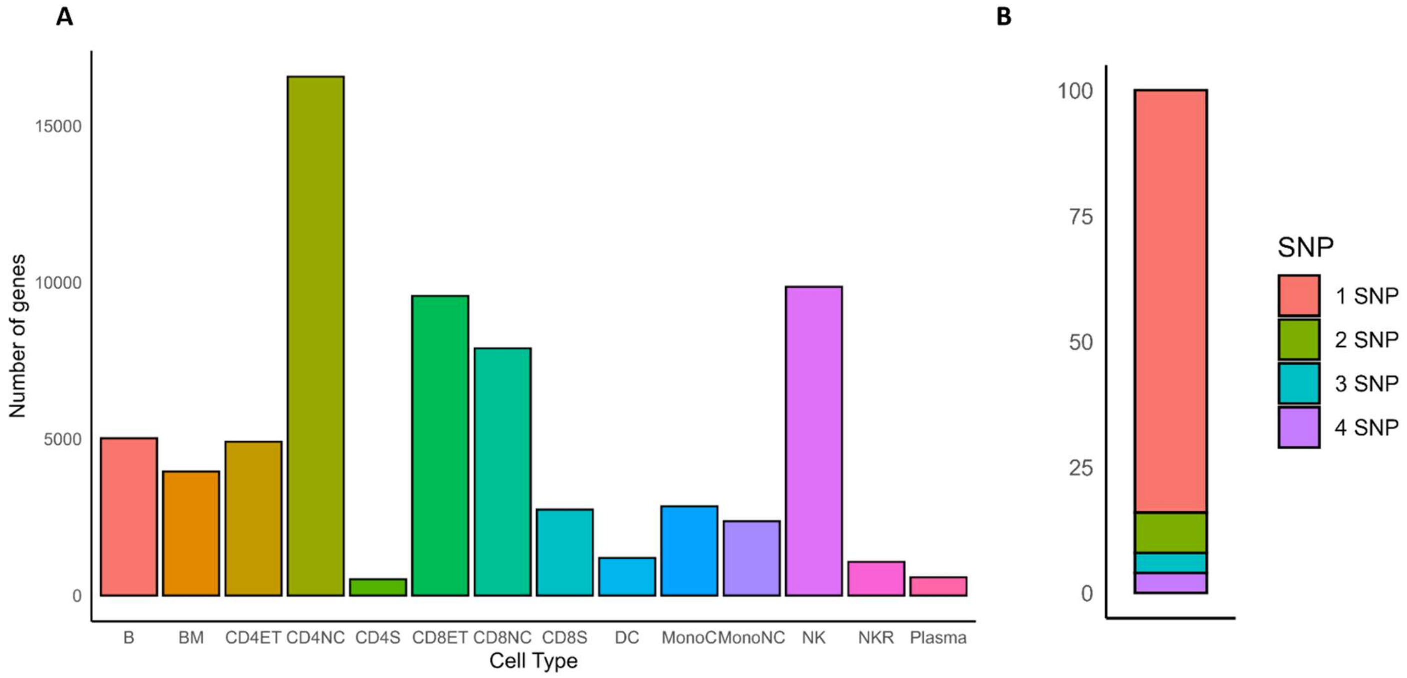

Figure S2 Stacked bar graph of the number of (a) eQTLs (b) genes unique to single-cell analyses

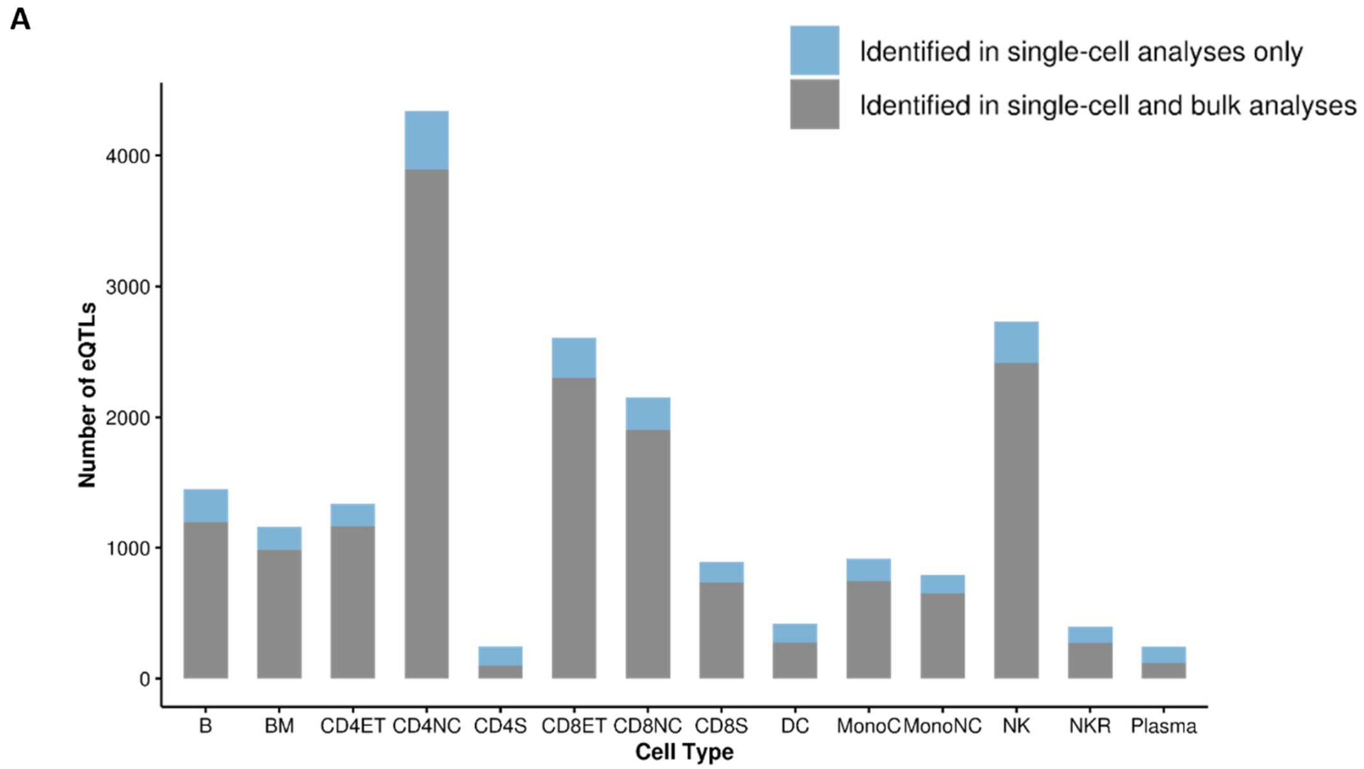

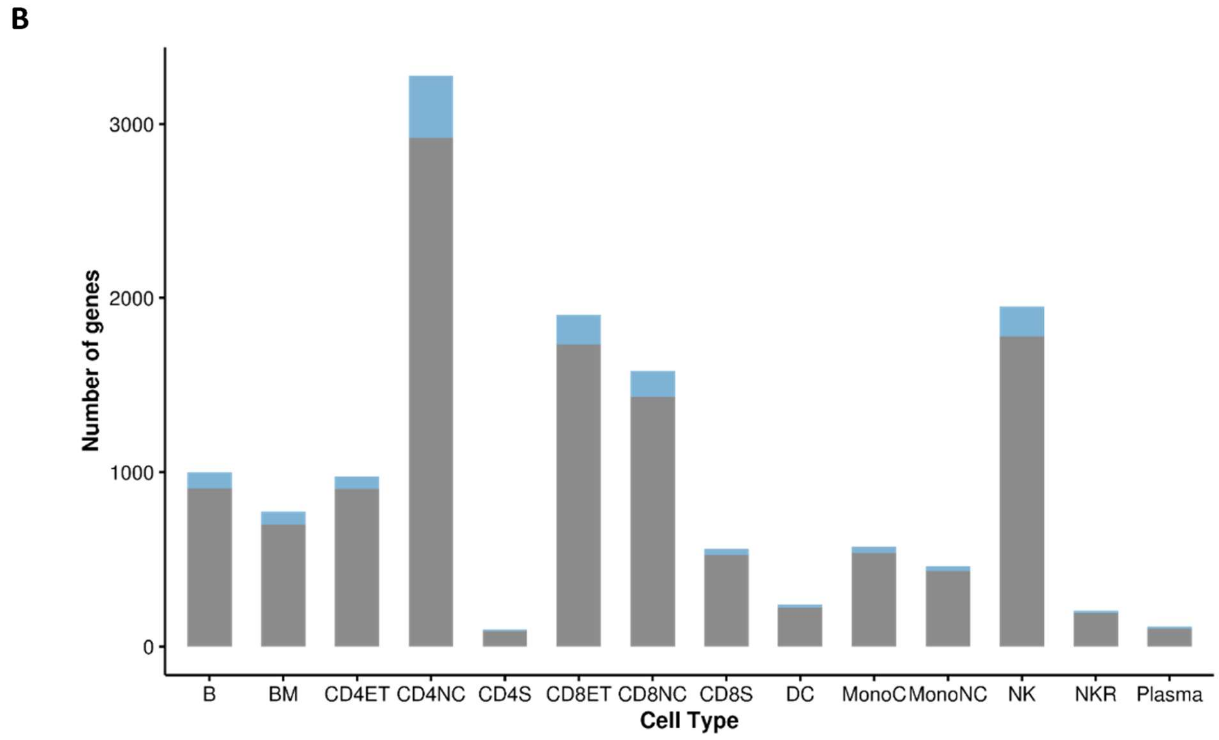

Figure S3. Uniform Manifold Approximation and Projection (UMAP) of scRNA-seq data of 15 human atherosclerotic plaque samples.

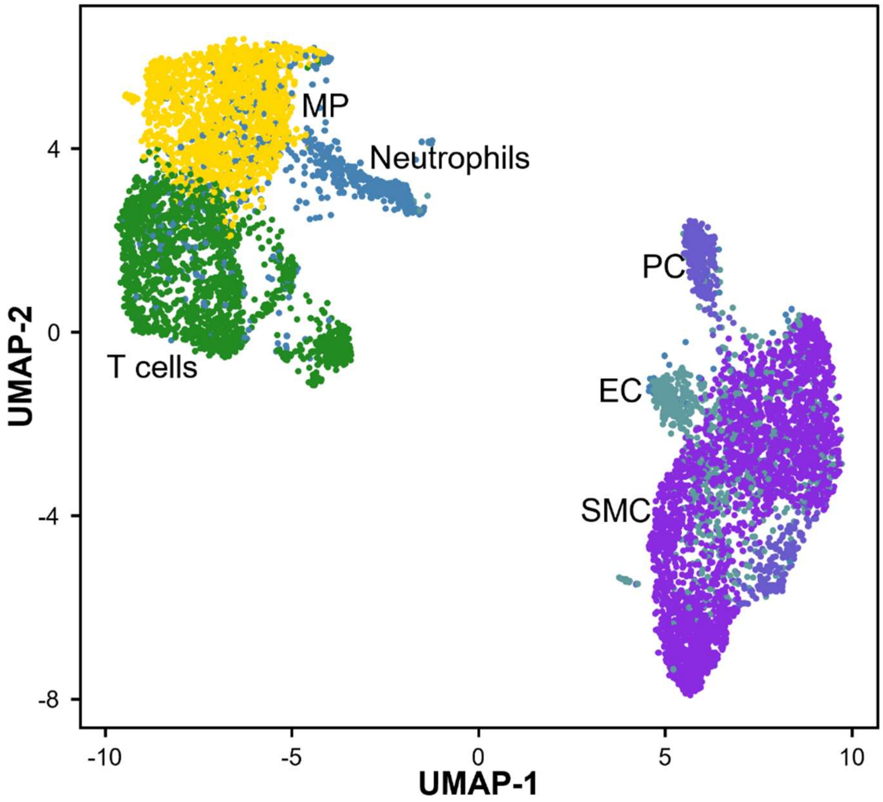

Figure S4. LIPA in macrophages (CD68) in human carotid plaque sections from symptomatic patients (n =3).

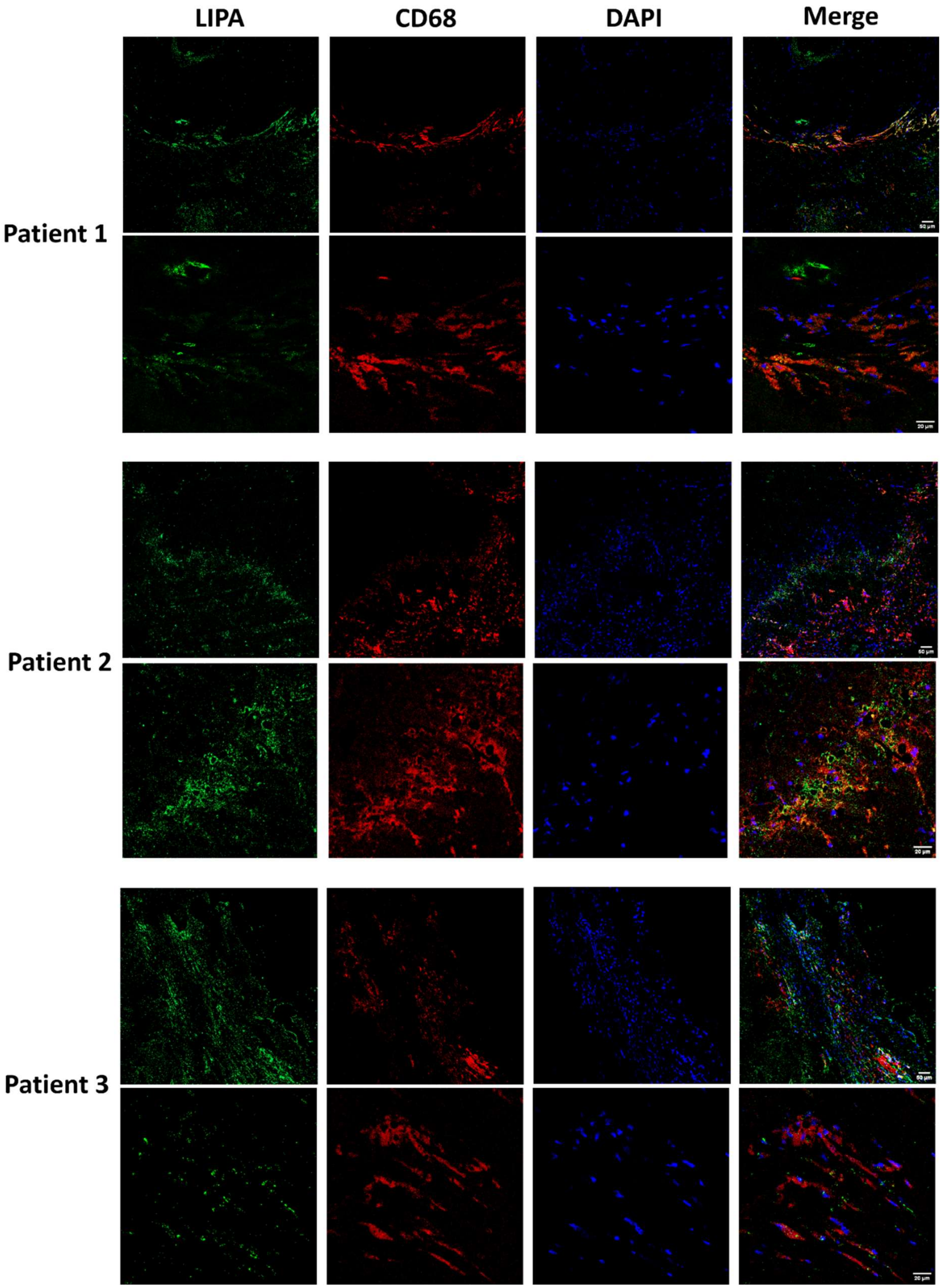

Supplement: Document S1. Figures S1–S4 [file mmc1.pdf]
